# Participation in EHR based simulation improves recognition of patient safety issues

**DOI:** 10.1186/1472-6920-14-224

**Published:** 2014-10-21

**Authors:** Laurel S Stephenson, Adriel Gorsuch, William R Hersh, Vishnu Mohan, Jeffrey A Gold

**Affiliations:** Department of Pulmonary and Critical Care Medicine, Oregon Health and Science University, 3181 SW Sam Jackson Park Rd, Portland, OR 97239 USA; Department of Medical Informatics & Clinical Epidemiology, Oregon Health and Science University, 3181 SW Sam Jackson Park Rd, Portland, OR 97239 USA

## Abstract

**Background:**

Electronic health records (EHR) are becoming increasingly integrated into the clinical environment. With the rapid proliferation of EHRs, a number of studies document an increase in adverse patient safety issues due to the EHR-user interface. Because of these issues, greater attention has been placed on novel educational activities which incorporate use of the EHR. The ICU environment presents many challenges to integrating an EHR given the vast amounts of data recorded each day, which must be interpreted to deliver safe and effective care. We have used a novel EHR based simulation exercise to demonstrate that everyday users fail to recognize a majority of patient safety issues in the ICU. We now sought to determine whether participation in the simulation improves recognition of said issues.

**Methods:**

Two ICU cases were created in our EHR simulation environment. Each case contained 14 safety issues, which differed in content but shared common themes. Residents were given 10 minutes to review a case followed by a presentation of management changes. Participants were given an immediate debriefing regarding missed issues and strategies for data gathering in the EHR. Repeated testing was performed in a cohort of subjects with the other case at least 1 week later.

**Results:**

116 subjects have been enrolled with 25 subjects undergoing repeat testing. There was no difference between cases in recognition of patient safety issues (39.5% vs. 39.4%). Baseline performance for subjects who participated in repeat testing was no different than the cohort as a whole. For both cases, recognition of safety issues was significantly higher among repeat participants compared to first time participants. Further, individual performance improved from 39.9% to 63.6% (p = 0.0002), a result independent of the order in which the cases were employed. The degree of improvement was inversely related to baseline performance. Further, repeat participants demonstrated a higher rate of recognition of changes in vitals, misdosing of antibiotics and oversedation compared to first time participants.

**Conclusion:**

Participation in EHR simulation improves EHR use and identification of patient safety issues.

**Electronic supplementary material:**

The online version of this article (doi:10.1186/1472-6920-14-224) contains supplementary material, which is available to authorized users.

## Background

The use of electronic health records (EHR) continues to grow in the US. The reasons are myriad, and include financial incentives related to the American Recovery and Reinvestment Act as well as a body of literature suggesting benefits of EHR use such as improved safety, improved efficiency and increased adherence to guideline based care [1–3]. The passage of the Health Information Technology for Economic and Clinical Health (HITECH) in 2009 has promoted the adoption of EHRs, and incentivization of EHR meaningful use has greatly expanded the role of EHRs in acute care hospitals [4]. From 2008 to 2012, use of any EHR in US hospitals increased from 9.4% to 44.4% and the percent of hospitals adopting a comprehensive EHR (defined as a system that includes electronic patient demographics, computerized provider order entry, results management and decision support) has risen from 1.6% to 16.9% over the same time period [5].

As EHRs become increasingly prevalent in healthcare, we continue to see a number of unintended consequences associated with their use. In 2005, Han et al. described the implementation of a computerized provider order entry (CPOE) system in a pediatric ICU. During the post-implementation study period they noted an increase in mortality associated with a change in ICU workflows [6]. There are a multitude of reasons for reports such as these, many of which were recently described in an Institute of Medicine Report on EHR safety. Specifically, the authors detail the impact of poor EHR implementation, inadequate training and education and the impact of the EHR user interface on inducing cognitive errors in medical decision making [7]. This last component is closely related to the contextual nature of the data generated, entered into, and viewed in the EHR. These issues are magnified in the ICU, as an individual patient generates more than 1400 data items per 24 hour period (excluding clinical notes, medication orders and details of medication administration) and could explain why many of the reports of difficulty with implementation of the EHR have come from the ICU environment [6, 8, 9].

With this increasing awareness of the potential negative impact of poor implementation and/or use of EHRs, there has been greater attention paid towards integrating the EHR into medical education and defining the competencies associated with proficient use [10–12]. Unfortunately, most EHR education activities are perceived to be inadequate by most providers. In one study looking at 9 basic EHR competencies, medical interns were unable to complete these tasks without assistance between 7 and 37% of the time, depending on the competency [11]. In another study, authors noted at least 3–5 days of training is required for physician satisfaction with the EHR and usability continued to improve even after a full week of training [13]. Given the time constraints placed on individual practitioners, it is unreasonable to expect that this amount (3–5 days) of training can be universally implemented. Further, most EHR training programs are generic and often not specific tailored to individual workflows. Combined, these issues suggest other approaches are necessary to successfully affect EHR implementation and training.

Simulation is increasingly used as a modality in physician training in large part due to several benefits such as posing minimal risk to patients, allowing for standardization of training environments and tailoring clinical situations to meet the needs of learners. The most established role for simulation in medicine has been to improve user proficiency with highly complex medical devices and procedures such as ultrasound, angiography and laparoscopic surgery with multiple studies documenting transfer of skills from the simulation suite to the clinical environment [14–16]. A growing number of studies demonstrate that simulation based educational activities can also improve the ability of subjects to manage medical emergencies and improve diagnostic (cognitive) performance when subjects subsequently encounter similar patients in real life [17–19]. *In toto*, it appears that utilizing a curriculum that includes simulation training improves subjects’ readiness for acute care in the inpatient setting [20]. Due to all of these benefits, and the complexity of the EHR, many groups including the IOM and the National Institute of Standards and Technology (NIST) recommend the use of simulation to aid in EHR education [21, 22]. There are few previous reports of the use of simulation to improve the use of the EHR as an adjunct to initial EHR training. In one study, investigators were successful in creating a realistic simulated ICU environment including use of an EHR to test decision-making variability in patient triage [23]. However, in most simulation-centered studies, the EHR was utilized as a tool as opposed to the focus of the simulation exercise itself. The few other studies exclusively centered on EHR simulation have not been in the ICU nor have they tested physician ability to recognize and process information (as opposed to order entry) [24, 25].

We recently published our preliminary experience with creation of an EHR based simulation exercise in which high-fidelity, data rich cases were created specifically to test whether participants could identify patient safety related issues and/or changes in clinical status. We demonstrated that the average user identified only a fraction of the issues present within the case and that their degree of patient safety issue or error recognition was independent of training level [26]. In this report, we describe the creation of an additional case with similar performance characteristics and the subsequent use of this case in our simulations with immediate debriefing. This can be used as a viable method of training that allows a significant transfer of learning effect through participation in the EHR simulation.

## Methods

The study was approved by the Oregon Health and Science University Institutional Review Board. The study was deemed minimal risk and formal informed consent was not required, however all participants were provided with an information sheet about our research protocol.

We have previously published our creation of a simulated patient environment within our EHR, case creation and the detailed method of the simulation exercise for evaluating use of the EHR in the ICU [26]. Based on the initial results, we created a second simulated medical ICU (MICU) patient with a different clinical scenario, different trends in vital signs and lab values, and incorporated new patient safety issues. The two cases were similar in the number of patient safety issues/action items to be identified, and the types of EHR skills required for completion, including data finding and assessment of trends. In both cases we attempted to make the EHR data as robust as possible, with hourly vital signs, intake and output reports, lab data, as well as resident, attending, nursing and respiratory therapist notes. Prior to using the second case as part of our simulation testing, we had several trainees run through the simulation to ensure compatibility. A complete list of errors included in each case as well as the type of error represented is listed in the Additional file 1: Table S1.

Subjects participated in the EHR simulation while on service in the MICU. Testing occurred once per week as previously described [26]. Subjects were given a written signout and were then allowed ten minutes to review the EHR. Participants then presented the case to a member of the study team and were graded on the number of patient safety issues identified. After the exercise, every participant underwent an immediate, standardized debriefing session on action items missed and received suggestions to improve their skills for EHR use. Beginning with the laboratory data, participants were shown the important trends in renal function and blood counts, as well as a tutorial regarding the graphing functions available. From there, assessment and evaluation of the medication administration report was completed, with discussion of appropriate dosing of medications and finding therapeutic drug monitoring assessments. This would be followed by reviewing vital signs, beginning with the most commonly used screen to assess vitals and using two other screens that display the same information in different contexts. Participants were shown possible customizability options and graphing functions within the vital signs pages as well as specific information found only in these screens. Next, participants would review ventilator data and discuss lung protective and low tidal volume ventilation, as well as how to assess appropriateness of an individual patient’s ventilator settings. Volume status and intake/output reports were then viewed and specific issues surrounding volume status in ARDS were discussed. Finally, participants were given time to ask questions, re-review any functions of the EHR, and discuss any concerns regarding participation in the simulation exercise. Participants were tested initially with either Case #1 or Case #2, with cases rotating on a weekly basis to ensure an equal distribution of participants in each case.

In order to test the effect of participation in the EHR simulation on performance, a cohort of 25 subjects were enrolled twice based on their presence in the ICU on the day of testing. When subjects underwent repeat testing, we ensured that they were tested with a different case from their initial test. Note one participant was initially served as a beta-test subject for the development of Case #1; as such only his performance on repeat testing is included.

We used a non-paired T-Test to compare performance among first time test takers for each case. To evaluate the effect of repeat testing for individual participants, we used a paired analysis. A chi square test was used to assess performance on individual metrics for each case. All data was analyzed with Graphpad Prism (La Jolla, CA).

## Results

We enrolled 116 subjects in our study, all of whom rotated through the Medical ICU at our institution. Among first time participants in the simulation, we enrolled 55 interns, 35 residents and 26 fellows. Of these, 71 subjects were tested with Case #1 and 45 subjects were tested with Case #2. Note that data on the first 39 subjects to participate in the simulation using Case #1 were previously published [26].

**Figure 1:**
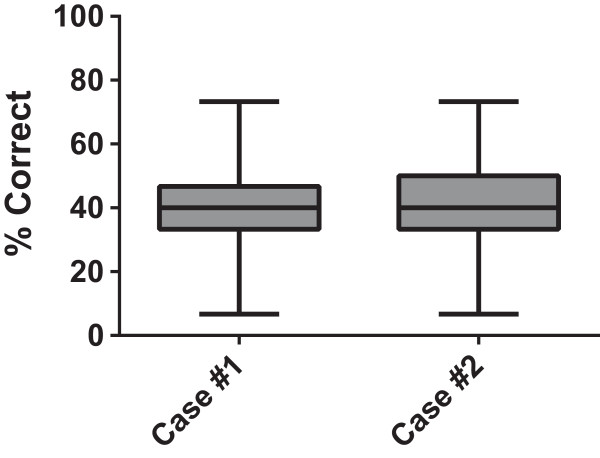
**Case 1 and Case 2 have equal performance characteristics among first time test takers.** % of errors recognized for first time participants for Case #1 (N = 71) and Case #2 (N = 49).

For first time participants, there was no difference in the percent of patient safety issues recognized between Case #1 (39.5%) and Case #2 (39.4%) demonstrating relative equivalency of difficulty of the two cases (Figure 1). For individual safety issues within both cases, there was an equivalent distribution of error recognition for each action item with the majority (85%) of items within the 2 cases recognized between 20 and 80% of the time by participants (not shown). This not only exhibits the comparable nature of the difficulty between the two cases, but also demonstrates that the lack of consistency in recognition of issues within the case is dependent on the participant as opposed to the case.

**Figure 2:**
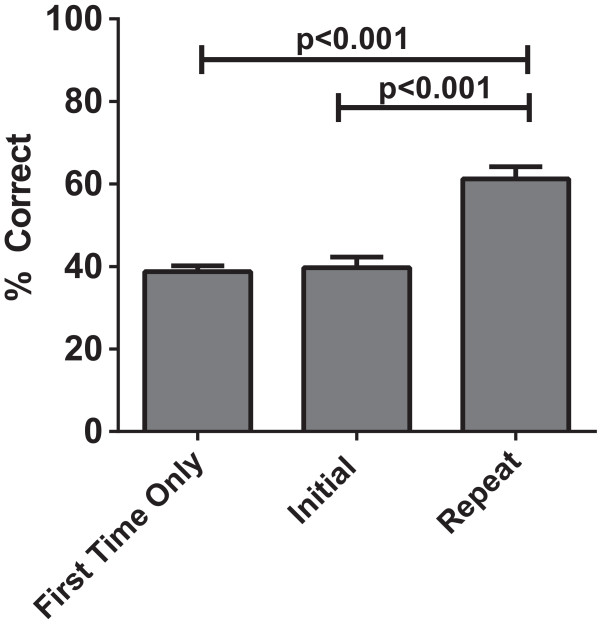
**Subjects participating in repeat testing have similar baseline performance.** 25 subjects participated in repeated testing. Their baseline performance in the simulation was identical to those who did not participate in repeat testing (N = 91).

Twenty-five subjects underwent testing with both cases. At the time of repeat testing, the cohort comprised of 18 residents (4 of whom were interns) and 7 fellows. For those who participated in repeat testing, their initial performance on Case #1 and Case #2 was no different than those who only participated in the simulation once (Figure 2). Of those participants, 16 were initially tested with Case #1 followed by Case #2 and 8 were first tested with Case #2 followed by Case #1. The interval between testing was greater than 4 weeks for 20 of the repeat participants (indicating a different ICU rotation), with a maximum interval of less than 1 year. Five of the participants underwent repeat testing after less than 4 weeks, with a minimum interval of 1 week between testing sessions for all subjects. Of these participants, four of the five underwent repeat testing one to two weeks after initial participation.

**Figure 3:**
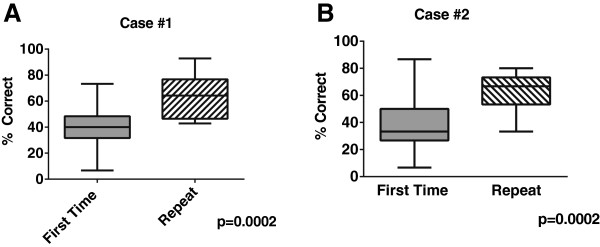
**Repeat test takers perform better than first time test takers for each individual case. Panel A**- Performance for first time participants for Case #1 (N = 71) and repeat participants (N = 8) (who were initially trained on Case #2). **Panel B**. Performance for first time participants for Case #1 (N = 45) and repeat participants (N = 17) (who were initially trained on Case #2.

For subjects repeating the simulation, their scores on repeat performance were greater than the performance for first time only participants (62% vs. 39%) (Figure 2). This persisted irrespective of level of training (62% vs. 30% (Interns), vs. 42% (Residents), vs. 49% (Fellows)). When we analyzed data by case, subjects who participated initially with Case #2 then went on to view Case #1 correctly identified 64.3% of action items in Case #1 (their second case viewed) as opposed to first time participants who correctly identified 39.5% of action items in Case #1, a 38.5% relative improvement (p = 0.0002). We observed similar data with Case #2, in that repeat participants identified 68.6% of action items compared to 39.4% among first time viewers, a 42.6% relative improvement (p = 0.0002) (Figure 3B & C).

**Figure 4:**
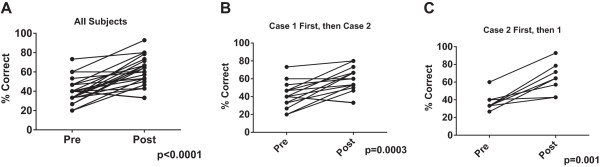
**Individual performance improves with participation in simulation. Panel A**. Initial and repeat performance for all individuals (N = 25). **Panel B**. Initial and repeat performance for subjects who started with Case #1 (N = 17). **Panel C**. Initial and repeat performance for subjects who started with Case #2 (N = 8).

**Figure 5:**
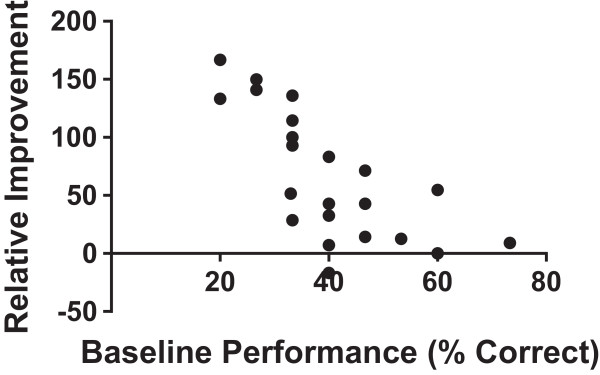
**Relative improvement in performance correlates inversely with baseline performance.** Correlation between relative improvement in simulation and baseline performance (R = -0.69; p = 0.002).

When we analyzed data by controlling for individual baseline performance, we observed a similar overall affect. For the entire cohort we found that individual subjects improved their rate of overall recognition of patient safety issues when re-tested with a different case (39.9% vs. 63.4%, p < 0.0001, Figure 4A). This effect remained regardless of whether the subject underwent simulation first with Case #1 followed by Case #2 (41.5% vs. 62.8%, p = 0.0003) or with Case #2 followed by Case #1 (37.5% vs. 64.3%, p = 0.001) (Figures 4B and C). When we looked for predictors of magnitude of improvement, there was no difference in relative improvement between residents and fellows or whether testing sessions occurred <4 weeks apart or >4 weeks (Data not shown). There was a strong correlation between baseline performance and magnitude of improvement, with poor performers showing the greatest relative improvement (Figure 5).

**Figure 6:**
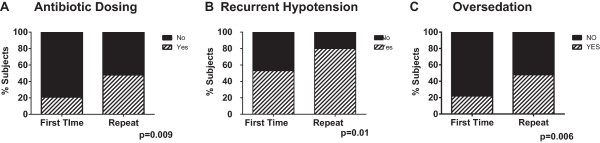
**Repeat test takers perform better in identification of specific safety issues.** **Panel A**. Recognition of misdosing of antibiotics among first time and repeat participants. **Panel B**. Recognition of new hypotension among first time and repeat participants. **Panel C**. Recognition of inappropriate MASS score.

While the content of the two cases was different, there were 3 core themes common to both cases. First was recognition of inappropriate medication dosing based on renal function. When combining data from both case progression scenarios (Case #1 followed by Case #2, and vice versa), only 21% of first time participants recognized the inappropriate dosing of antibiotics compared to 48% among repeat test-takers (p < 0.006) (Figure 6A). Second was ability to recognize significant changes in hemodynamics over a period for >24 hrs. Again, only 53% of first time test-takers recognized these trends, which increased to 78% among repeat test-takers (p = 0.01) (Figure 6B). Third was recognition of oversedation based on Motor Activity Assessment Scale (MAAS). Again, repeat participants had a higher rate of recognition compared to first time participants (47 vs 22%; p < 0.009) (Figure 6C).

## Discussion

We created a novel EHR based simulation exercise to evaluate the use of the EHR by medical trainees at our institution. Our prior study suggested that residents and fellows do not adequately recognize clinical trends and other action item data within the EHR that are key to optimizing patient safety [26]. We have now expanded upon those findings with the creation of a similarly-calibrated second case with respect to rates of recognition of patient safety issues among first time test takers. As with the first case, there is a random distribution of recognition across the individual action items contained within Case #2. The creation of our second case allowed for repeat testing of prior participants, thus testing the effectiveness of the simulation as a learning activity.

The most important finding in our study is that identification of patient safety issues improved with repeated simulation. This effect is most likely due to participation in the simulation itself, as opposed to increased use of system in the period between tests, as repeat participants outperformed all first time only participants irrespective of level of training. Further, we found an improvement in recognition even after controlling for the case employed, both in general as well as in specific patient safety issues that were built into our simulation cases. Paired analysis indicated that the improvement in recognition of patient safety issues was independent of which simulated case was viewed first by the participant. While this finding is consistent with other studies indicating improvement in performance following use of simulation based training [27–29], prior research has focused on traditional simulation scenarios such as cardiopulmonary arrest training or obstetrics deliveries [27, 28]. Our study is the first, to our knowledge, confirming this finding using EHR in a high-fidelity case simulation setting.

In our study, repeat testing sessions were at least a week apart and most were more than a month apart, which suggests that participation in the simulation has a lasting learning effect on the subject. This is particularly emphasized by the process of debriefing that we employed, which fulfilled the structural elements required for the process as outlined by Lederman [30]. Traditional models of debriefing focus on identifying the impact of the activity, clarifying concepts, emotions, empathy, and engaging in systematic reflection and analysis [31–33]. In our study we specifically focused the debrief on optimizing the subjects’ ability to recognize critical data within the EHR and to optimize strategies for data finding and visualization. This short-duration process appears to have a lasting beneficial effect. More importantly, the degree of improvement in the simulation was inversely related to baseline performance, irrespective of level of training. This suggests that this exercise has its greatest benefit with those who have the greatest difficulty in using the EHR for effective clinical decision making.

In order to maximize the potential this exercise would have on reducing medical errors in the ICU, we incorporated 3 core areas into both cases: recognition of dangerous trends in hemodynamics, medication misdosing and recognition of oversedation. Multiple studies suggest failure to recognize problems in these 3 areas is associated with missed clinical deterioration including cardiac arrest, increased rate of medical errors (e.g. medication errors, missed diagnosis), increased time on the ventilator, and increased ICU length of stay or increased mortality [34–36]. For all 3 domains, repeat test takers consistently outperformed first time test takers. This has significant implications for both learning and patient safety. It also emphasizes the ability of this type of educational activity to address multiple different competencies simultaneously including Medical Knowledge, System Based Practice and Practice Based Learning as defined by the Accreditation Council for Graduate Medical Education (ACGME) [37]. Further studies will be required to determine if these exercises have any impact on rates of actual errors within the ICU or other care environments.

The ability to document a significant learning effect with our EHR based simulation now opens the possibility to incorporating the EHR into other high fidelity simulation activities. Our data amongst first time participants highlights the importance data acquisition from the EHR plays into clinical decision making. Thus, it will be essential to now incorporate the EHR routinely into other complex simulation scenarios in order to truly understand the role the EHR plays in this context. This becomes even more important for team based training. It is well established that not only do different professions utilize the EHR differently, but that the EHR can have independent effects on interprofessional communication [38]. Our use of high fidelity cases which contain relevant information for all members of the interprofessional team will allow for further expansion and testing of these learning activities.

Our study has several limitations. Most importantly, we are unable to identify whether the improvement in performance is due to improved utilization of the EHR or whether training with the EHR improved cognitive processing of information already viewed. While the overall net improvement in performance is perhaps the most relevant endpoint, especially in relation to patient safety, identifying the contribution of EHR skill acquisition vs. improved cognition will be essential for refinement of the activity. Our ability to create multiple standardized cases will allow us to incorporate more objective measures of EHR usability (such as eye tracking) into future simulations and is the subject of ongoing studies. Further, while the overall rate of recognition of patient safety issues improved from the first simulation to the second, we did not approach 100% recognition with repeat simulation. One likely factor is that each subject may require differing amounts of training to achieve optimal recognition rates, which may in turn be based on their level of clinical proficiency as well as their EHR navigation and use skill levels. This may be further confounded by subjects’ prior exposure to different EHRs before using the EHR at our institution. However, even if we are able to control for baseline EHR exposure, we do not know at this time how advantageous additional simulation exposure will be with respect to improving recognition of patient safety issues. We are creating additional cases to allow for continued participation in multiple simulation case scenarios to assess at what level, if any, there is a saturation effect to the benefit for participation in the exercise. It is possible that we will be unable to further improve performance with simulation training alone, especially if persistent inability to recognize patient safety issues, or a plateau in the detection rate, reflect issues related to the EHR user interface as opposed to clinician training.

Another issue is the fidelity of the activity in regards to provider workflow. For the simulation, subjects utilized an EHR interface identical to what they use clinically (including their unique customizations) in order to closely mimic their real-world EHR experience. Additionally, we conducted our simulations in the ICU at a dedicated work station to approximate the working environment of our participants. However, the simulations were conducted in isolation rather than as part of a larger workflow (as in pre-rounding) on a full complement of 5 or 6 ICU patients (for a given resident). Participants may also exhibit some degree of fatigue in the afternoons, when our simulations were conducted, since they would have already participated in a full day of rounds, a factor which may which may affect clinical their information processing and decision making. Indeed, there appears to be an association with resident fatigue on clinical decision making and medication errors in the ICU [39]. We did not maintain a record of the ICU census on each specific day of testing, which may limit our ability to assess the correlation between error detection rates and subject workloads on the day of simulation. Finally, we acknowledge that while performing simulations *in situ* might better recapitulate the real life use of the system, this may also have increased the likelihood of distractions and thus affected the ability of subjects to identify errors.

Many of our residents use pre-populated (auto-templated) electronic note templates as their rounding tool, which we did not make available in the simulation. As a result, the residents and fellows would hand write data to be presented. This difference in pre-rounding workflow may have affected their cognitive processing either positively or negatively as the generation of a rounding artifact can affect information processing [40, 41]. In addition, during patient care rounds, the resident does not present data in isolation, rather there are other members of the inpatient team, such as nursing staff, attending physicians, and pharmacists who contribute to the daily plan. We do not know how many of the errors or action items missed by the subject would have been subsequently recognized by other members of the interprofessional rounding team. This will be further assessed as part of a future study involving interdisciplinary teams in our simulation exercise.

## Conclusion

In conclusion, we have created a novel simulation environment for evaluating the use of the EHR in a clinical environment and have found that we are relatively poor at recognizing patient safety issues and trends within the EHR. We demonstrated overall improvement in identification of patient safety issues with repeat high-fidelity clinical case-based simulation, however we note that identification rates improve primarily in specific areas such as the evaluation of longitudinal trends and medication errors, but remain poor in other areas. We are continuing to evaluate how residents and fellows use the EHR to evaluate data and what strategies may be more effective in improving patient safety in the ICU.

## Electronic supplementary material

Additional file 1: Table S1: Patient Safety Issues for both cases. (DOCX 14 KB)
