# Supplementary material for: Participation in EHR based simulation improves recognition of patient safety issues
Source: BMC Med Educ. 2014 Oct 21;14:224. doi: 10.1186/1472-6920-14-224 (PMC4287422; doi:10.1186/1472-6920-14-224)
Supplement: Supplementary file 1 — Additional file 1: Table S1: Patient Safety Issues for both cases. (DOCX 14 KB) [file 12909_2014_1053_MOESM1_ESM.docx]

| **Case #1** | **Safety Issue Category** | **Case #2** |
| --- | --- | --- |
| **Change in patient condition** |  | **Change in patient condition** |
| 25% drop in mean arterial pressure and increase in Heart rate | Error of Cognition  Customization, structure and time | 30% decrease in Hematocrit |
| Recurrent sepsis | Error of Cognition | Recurrent Hypotension |
| Increasing plateau pressure >30 | Data Finding, overcompleteness | Hypernatremia |
| Increase in WBC | Structure and time, cognition | Developing Lactic Acidosis |
| New fever | Structure and time, cognition | Platelets decreased by 50% |
| **Medication Errors** |  | **Medication Errors** |
| Inappropriately low antibiotic dose based on renal function | Data finding, cognition | Low Molecular Weight Heparin dose inappropriately high |
| Low antibiotic trough | Data finding, cognition | Ongoing use of lisinopril |
| Use of D5W in hyperglycemic patient | Data finding, overcompleteness | Continued on outpatient Metformin |
|  | Data finding, overcompleteness | Continued on long-acting insulin despite hypoglycemia |
|  | Data finding, cognition | Inappropriately high dose of antibiotic based on renal failure |
| **Failure to Adhere to Best Practice Guidelines** |  | **Failure to Adhere to Best Practice Guidelines** |
| Glucose >200 | Data finding, overcompleteness | Glucose <80 |
| Tidal volume of 8 mL/kg IBW in Acute Respiratory Distress Syndrome | Data finding and cognition | Positive End Expiratory Pressure inappropriately high based on fraction of inspired oxygen |
| Over Sedation * | Data finding | Over Sedation* |
| Lack of Daily Awakening | Data finding | Inappropriate Head of Bed order |
| Positive fluid balance | Data finding | Lack of GI prophylaxis in intubated patient |
| Inadequate tube feeding | Data Finding, overcompleteness | Inadequate tube feeding |

*Based on Motor Activity Assessment Scale (MAAS)

**Patient Safety Issues for both cases.**
